# Field-controlled ultrafast magnetization dynamics in two-dimensional nanoscale ferromagnetic antidot arrays

**DOI:** 10.3762/bjnano.9.104

**Published:** 2018-04-09

**Authors:** Anulekha De, Sucheta Mondal, Sourav Sahoo, Saswati Barman, Yoshichika Otani, Rajib Kumar Mitra, Anjan Barman

**Affiliations:** 1 Department of Condensed Matter Physics and Material Sciences, S. N. Bose National Centre for Basic Sciences, Block JD, Sector III, Salt Lake, Kolkata 700 106, India; 2 Institute of Engineering and Management, Sector V, Salt Lake, Kolkata 700 091, India; 3 Institute for Solid State Physics, University of Tokyo, 5-1-5 Kashiwanoha, Kashiwa, Chiba 277-8581, Japan; 4 RIKEN-CEMS, 2-1 Hirosawa, Wako, Saitama 351-0198, Japan; 5 Department of Chemical Biological and Macro-molecular Sciences, S. N. Bose National Centre for Basic Sciences, Block JD, Sector III, Salt Lake, Kolkata 700 106, India

**Keywords:** ferromagnetic antidot lattice, magnonic crystal, micromagnetic simulations, spin-wave modes, time-resolved magneto-optical Kerr effect

## Abstract

Ferromagnetic antidot arrays have emerged as a system of tremendous interest due to their interesting spin configuration and dynamics as well as their potential applications in magnetic storage, memory, logic, communications and sensing devices. Here, we report experimental and numerical investigation of ultrafast magnetization dynamics in a new type of antidot lattice in the form of triangular-shaped Ni_80_Fe_20_ antidots arranged in a hexagonal array. Time-resolved magneto-optical Kerr effect and micromagnetic simulations have been exploited to study the magnetization precession and spin-wave modes of the antidot lattice with varying lattice constant and in-plane orientation of the bias-magnetic field. A remarkable variation in the spin-wave modes with the orientation of in-plane bias magnetic field is found to be associated with the conversion of extended spin-wave modes to quantized ones and vice versa. The lattice constant also influences this variation in spin-wave spectra and spin-wave mode profiles. These observations are important for potential applications of the antidot lattices with triangular holes in future magnonic and spintronic devices.

## Introduction

Recent advances in nanofabrication techniques have resulted in artificially patterned magnetic metamaterials, known as magnonic crystals (MCs), which have great potential for technological applications and fundamental research [1–2]. Investigation and tailoring of the magnetization dynamics in ferromagnetic nanodots [3–4], nanowires [5] and antidots [6–8] have fuelled considerable research on reconfigurable MCs, which act as a media for standing and propagating spin waves (SWs) in the GHz frequency regime. Ferromagnetic antidot lattices (magnetic thin films with periodic non-magnetic inclusions or embedded holes) have emerged as one of the strongest candidates for reconfigurable, effective media for SW propagation due to the larger propagation velocity (steeper dispersion) than nanodot lattices. They find potential applications in magneto-photonic crystals [9], ultrahigh density data storage media [10], frequency-based magnetic nanoparticle detectors [11], waveguides for SWs [12–13], spin-wave filters [14], spin-logic [15] and reprogrammable magnonic devices [16]. The edges of the antidots lead to quantization of SW modes due to lateral confinement as well as the generation of a periodically modulated internal magnetic field due to the demagnetization effect. A number of parameters can be varied to tune the magnonic spectra and magnetization dynamics in ferromagnetic antidot lattices. Several studies have been focused on the engineering of the coercive field, magnetoresistance and anisotropy properties on domain formation and the magnetization reversal mechanism with the change of shape, size and density of antidots [17–18]. Extensive research on the dynamics of standing and propagating SWs in antidot lattices has shown pattern induced splitting [19], confinement, localization and propagation of SWs, depending upon the lattice and antidot geometry, base material and strength and orientation of the bias field [6–8,19–26]. Intrinsic configurational magnetic anisotropy arising due to the internal field variation can be tuned effectively by varying the antidot lattice symmetry [21,24]. The shape of the antidots is found to control the SW mode structures as well as the anisotropy in the frequency spectra [25]. Quantized SW modes have been found to be transformed to propagating ones and vice versa in rhombic antidot lattices with the variation of the in-plane orientation of the bias-magnetic field [26]. A particular study showed the hysteresis and anisotropy properties of Ni_80_Fe_20_ (a permalloy, noted as Py hereafter) antidot lattices with hexagonal symmetry by the influence of the hole size, lattice packing fraction and scale factor via micromagnetic numerical approach [27]. Bi-component or filled antidot lattices can tune the SW properties and magnonic band structure more efficiently due to the strong interelement exchange and dipolar coupling [28–29]. A remarkable difference in magnetic anisotropies and magnetization reversal mechanisms has been observed in systematically engineered square and binary antidot lattices [30].

Hexagonally arranged antidot lattices are interesting because they offer the highest packing density features among all Bravais and non-Bravais lattices. In contrast to other lattice symmetries, the hexagonal lattice structure exhibits six-fold anisotropy with an easy axis that alternates at every 60° and it does not obey the nearest-neighbour rule as the easy axes are oriented along the edges of the hexagonal unit cell [26,31–32]. All of those studies were performed on antidots of circular shape, and very rarely, antidot lattices with triangular-shaped holes, which may suffer from edge effects due to the sharp triangular edges of the holes, have been explored [25]. Unlike the circular or square-shaped antidots, the demagnetized regions around the sharp edges of the triangular-shaped antidots are asymmetric. These may lead to interesting properties of SW quantization in the regions between the antidots. Here, we have focused on the detailed and systematic investigation of the magnetization dynamics in two-dimensional Py antidot lattices where nanostructured triangular holes are arranged in a hexagonal lattice using an all-optical time-resolved magneto-optical Kerr microscope. We have investigated the variation in the nature of the extended and quantized SW modes in such systems by changing the strength and orientation of the in-plane bias-magnetic field and the lattice constant of the array. Micromagnetic simulations have also been performed to understand and interpret the experimental results, which helped to unravel the transformation of extended SW modes to quantized ones with the angular variation of the in-plane magnetic field and change in lattice constant. The opening and closing of channels for spin-wave extension and localization for a large number of bias field angles (in the full range of 0° to 90°) for this type of complex magnonic crystal have not been studied earlier. Also the sharp corners of the triangular holes of the high density antidot lattice and the complicated lattice structure create inhomogeneous internal magnetic fields due to the effective pinning centres for SWs created by the asymmetric demagnetized regions between the neighbouring triangular holes, which may give rise to some new and interesting physics of opening and closing of new channels for spin-wave extension and/or localization at particular edges of the triangular holes. Finally we have extensively studied the variation in the internal magnetic field profiles and demagnetizing regions for different angular orientation of the applied bias magnetic field and lattice constant for deeper understanding of the origin of the observed SW modes in such complex magnonic crystal, which was not done previously.

## Results and Discussion

**Figure 1:**
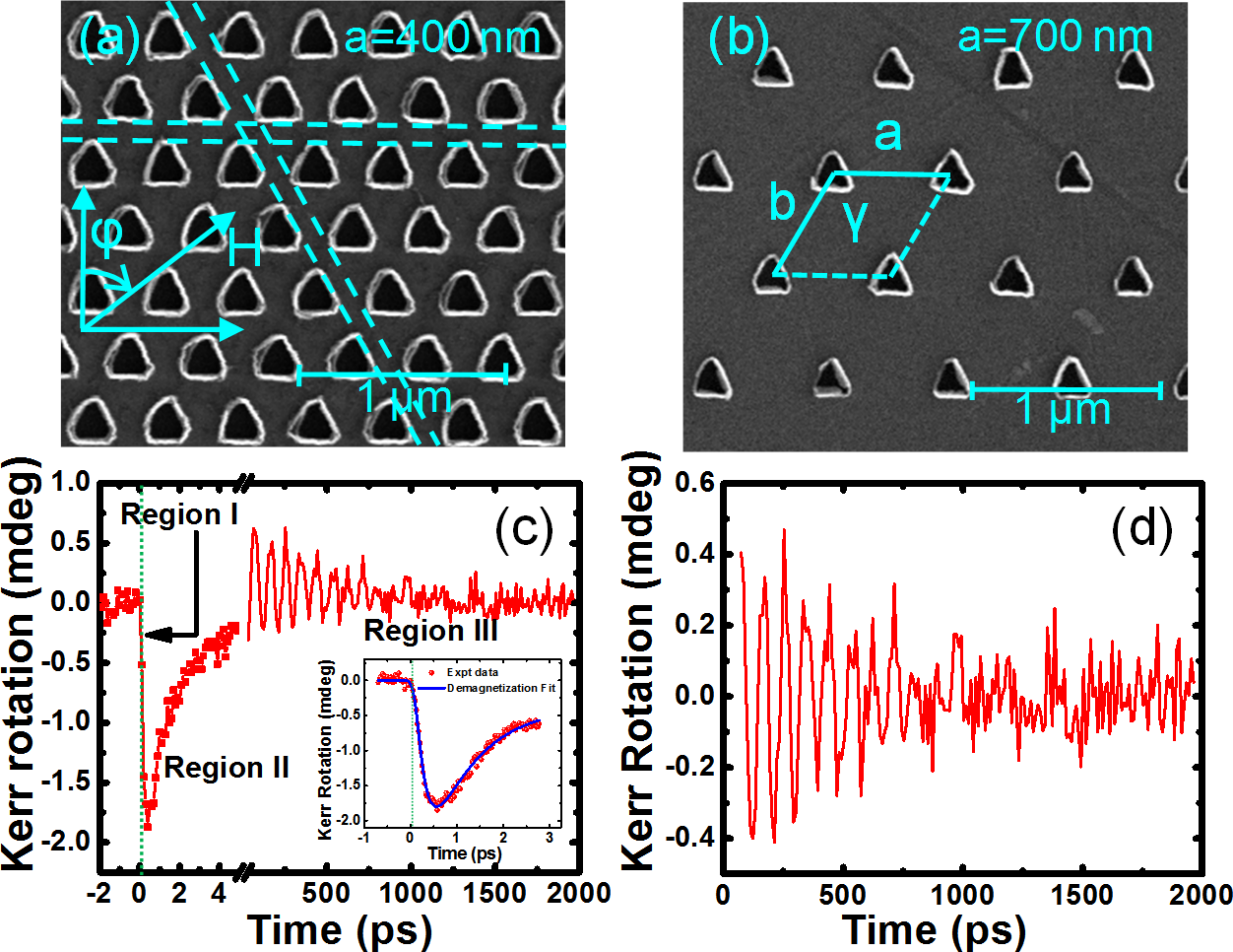
Scanning electron micrographs of the Py antidot arrays with triangular holes of edge length 200 nm, thickness 20 nm, arranged in hexagonal lattice with varying lattice constants, (a) *a* = 400 nm, *b* = 360 nm and (b) *a* = 700 nm, *b* = 560 nm. The lattice constants are shown in the micrographs along with the length scales. The unit cell is marked inside the lattice. The horizontal and diagonal channels for spin-wave propagation are marked by dotted lines and the applied bias field geometry is shown in (a). (c) Typical time-resolved Kerr rotation data for the array with *a* = 700 nm for *H* = 1.3 kOe. The inset shows the time-resolved Kerr rotation data for shorter time window obtained with a higher temporal resolution (symbols) and fit with a three-temperature model (solid line) for extraction of the ultrafast demagnetization and fast relaxation time. The zero delay is shown by the vertical dotted line. (d) Background-subtracted time-resolved Kerr rotation data showing the precessional oscillation of magnetization.

25 × 25 μm^2^ arrays of two-dimensional Py antidot arrays with triangular holes arranged in hexagonal lattice symmetry have been fabricated by a combination of electron-beam lithography, electron-beam evaporation and ion milling [20]. Figure 1a,b shows the scanning electron micrographs (SEMs) of the two hexagonal antidot arrays, S1 and S2. The edge length of the triangular holes is about 200 nm and the separation between the nearest edges for the two samples is about 200 nm and 500 nm, respectively (lattice constants (*a*) are 400 nm and 700 nm, respectively). About ±5% deviation in the edge length of antidots and lattice constant is observed. The SEM images show that the triangular antidots have rounded corners and they suffer from small asymmetry in their shapes. The above deviations and asymmetry in the shape of the antidots have been included in the micromagnetic simulations as described later in this article.

The lattice parameters *a* and *b* are varied while the angle γ is kept constant at 120° for the hexagonal lattice as shown in Figure 1b. The unit cell is also marked in Figure 1b. The values of *a* and *b* (in nm) as obtained from the SEM images are 400 and 360 and 700 and 560, respectively, for the two lattices. The value of γ is obtained as 120 ± 2°. For convenience, the antidot arrays will be described only by the lattice constant *a*, from here on.

The ultrafast magnetization dynamics was measured by using a home-built time-resolved magneto-optical Kerr effect microscope based upon a two-colour collinear pump–probe setup [33–34]. The second harmonic (λ = 400 nm, pulse width ≈100 fs) of a Ti:sapphire laser was used to pump the samples, while the time-delayed fundamental (λ = 800 nm, pulse width ≈80 fs) laser beam was used to probe the dynamics by measuring the polar Kerr rotation with an optical bridge detector. A variable amplitude magnetic field is applied to the sample, the direction of which was tilted slightly (10°) out of the plane of the sample to have a finite demagnetizing field along the direction of the pump pulse. The pump beam modulates this out of plane demagnetizing field to induce precession of magnetization in the sample. The in-plane component of the magnetic field is referred to as the bias magnetic field. The magnetization dynamics in the antidot arrays are measured as a function of strength (*H*) and angular orientation (φ) of the bias magnetic fields.

### Variation of magnetization dynamics with the variation of the orientation of applied bias magnetic field and lattice constant

Figure 1c shows representative time-resolved Kerr rotation data from the array with *a* = 700 nm with in-plane bias field of 1.3 kOe at φ = 0°. The graph reveals three important temporal regimes, namely, the ultrafast demagnetization (region I), fast relaxation (region II), and precessional motion superposed on a slow relaxation (region III). We have further performed precise measurements of the time-resolved Kerr rotation for about 3 ps from the zero delay with higher temporal resolution of 25 fs (Figure 1c inset) and fitted the data with the three temperature model using the analytical expression [35] given in Equation 1.

[1]
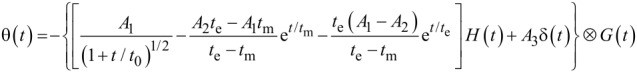


Here the time resolution for the laser profile is accounted by a Gaussian function *G*(t), and it is convoluted with the fit function, which contains two exponentials with time constants *t*_m_ and *t*_e_ representing the demagnetization and the fast relaxation time, respectively. *H*(*t*) and δ(*t*) represent the Heaviside step function and the Dirac delta function, respectively. A1, A2, and A3 are constants. From the fit we have obtained the ultrafast demagnetization time as 204 ± 3 fs and the fast relaxation as 1.0 ± 0.01 ps. This is followed by the slower relaxation process which occurs within 400 ± 7 ps, while the precessional oscillation is found to be superposed on the slower relaxation process. Figure 1d shows the precessional dynamics after removing the negative delay and ultrafast demagnetization and subtracting a bi-exponential background. Fast Fourier transform (FFT) is performed over this background-subtracted oscillatory Kerr rotation data to obtain the power vs frequency plot.

**Figure 2:**
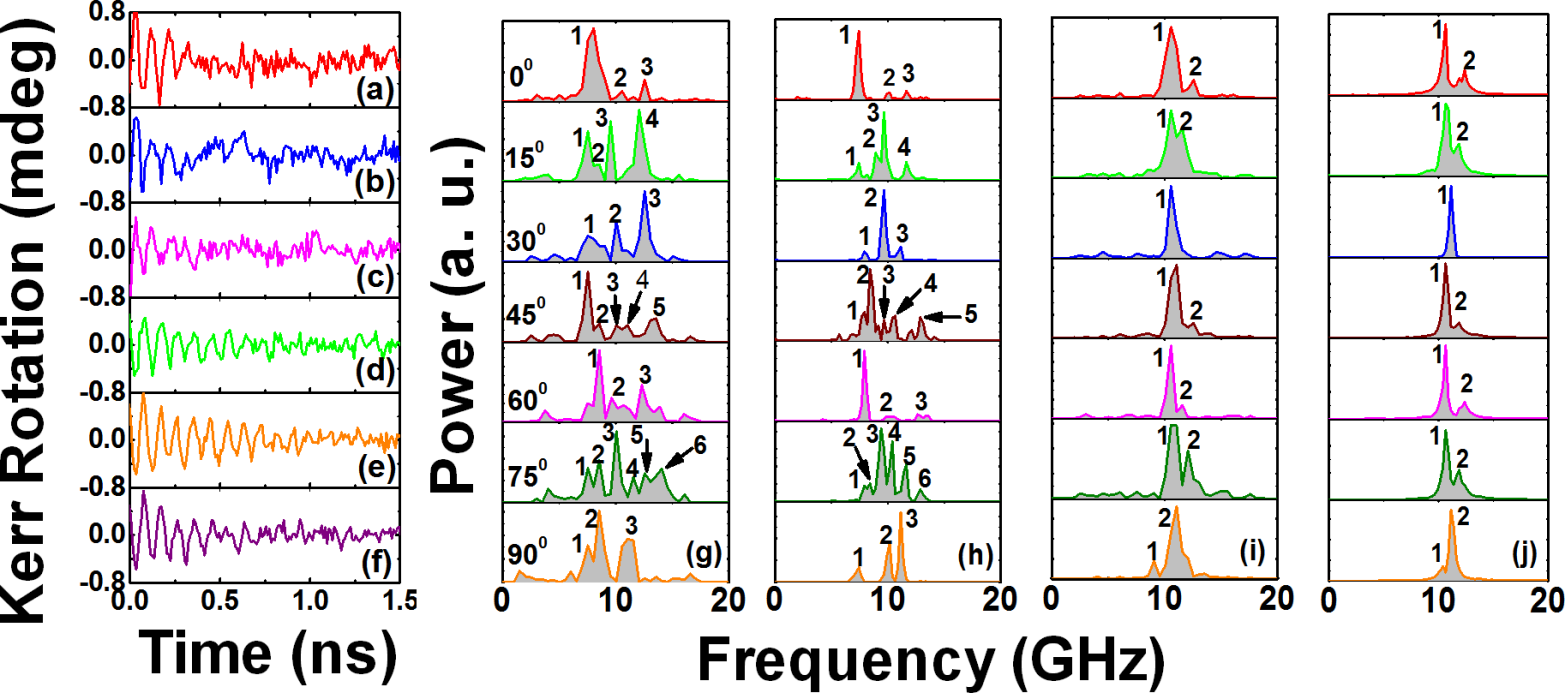
(a–f) Experimental time-resolved Kerr rotation data for some specific orientations of the bias magnetic field the two arrays: (a) S1 at 0°, (b) S1 at 45°, (c) S1 at 60°, (d) S2 at 0°, (e) S2 at 45°, and (f) S2 at 60°. (g) and (i) show the FFT power spectra of experimental time-resolved Kerr rotation data of S1 and S2 for different orientations of the in-plane bias field: (g) for S1 at *H* = 1.0 kOe and (i) for S2 at *H* = 1.3 kOe. (h) and (j) show the FFT power spectra of simulated time-domain magnetization: (h) S1 at *H* = 1.0 kOe and (j) S2 at *H* = 1.3 kOe. Mode numbers are shown in both experimental and simulated power spectra.

Figure 2a–f shows the representative background-subtracted experimental time-resolved Kerr rotation data for some specific orientations of the in-plane bias magnetic field for the two antidot arrays. The experimental FFT power spectra for S1 and S2 with φ varying from 0° to 90° (at an interval of 15°) are shown in Figure 2g and Figure 2i.

As φ and *a*-values are varied, we observe a distinct variation in the magnetization dynamics. For all φ and *a*-values, multimodal SW spectra are observed corresponding to the damped nonuniform oscillations [36].

The experimental data of bias-field-angle dependence of SW spectra for S1 has been taken at *H* = 1.0 kOe, whereas for S2 the data has been taken at *H* = 1.3 kOe. However, since both of the two field values are well above the saturation field for the Py (base material of the antidot lattice), the variation in bias field magnitude only changes the SW frequency values for the two samples, while the qualitative features of the angular dependence of SW spectra and corresponding SW mode profiles would not be affected by this. The experimental SW spectra for S1 show 3 modes for φ = 0°, 30°, 60° and 90°. The spectra for φ = 0° and 60° are qualitatively similar in nature (though the mode frequencies are not same). Also, there is a qualitative (but not quantitative) agreement between the spectra for 30° and 90°. However, the SW spectra are remarkably different for φ = 15°, 45° and 75° with a drastic increase in the number of modes at φ = 45° and 75°. These indicate a change in the collective nature of the magnetization dynamics with varying φ-values. When we consider S2 with larger *a*-values, we get a significant difference in the nature of SW spectra as opposed to S1. Here, instead of large number of modes, only two modes (one dominant mode with a low power shoulder) for almost all the angles are observed (excluding φ = 30° where instead of two, only one mode is observed).

The experimental SW spectra are well reproduced by micromagnetic simulations by OOMMF software [37]. The simulated FFT power spectra for S1 and S2 with the variation of φ are shown in Figure 2h and Figure 2j. As opposed to the experimental technique, which is based on optical excitation of magnetization, the simulation is performed by applying a pulsed magnetic field, which reproduces the experimental conditions successfully. The details of the simulation can be found elsewhere [4]. Similar simulation methods have also previously been used to successfully reproduce and understand the magnetization dynamics and SW mode profiles in different types of magnonic systems [34,36,38]. We have studied arrays of 7 × 7 antidots and discretized the arrays into rectangular prisms of dimensions 4 × 4 × 20 nm^3^. The excitation over the whole array is uniform and we have extracted the data from the centre of the arrays. The lateral cell size is well below the exchange length of Py (≈5.2 nm). The shapes introducing the actual edge roughness of the triangular holes have been derived from SEM images and the material parameters used for Py were gyromagnetic ratio γ′ = 17.6 MHz Oe^−1^, anisotropy field *H*_k_ = 0, saturation magnetization *M*_s_ = 860 emu cm^−3^, and exchange stiffness constant *A* = 1.3 × 10^−6^ erg cm^−1^. The material parameters were extracted by measuring the variation of precessional frequency (*f*) with bias magnetic field *H* for a Py thin film and by fitting them using Kittel formula,

[2]
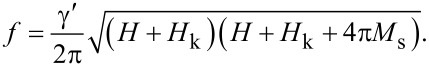


The exchange stiffness constant *A* is obtained from literature [39]. A pulsed field of peak value of 30 Oe, 10 ps rise/fall time and 20 ps pulse duration is used perpendicular to the sample plane, while a damping coefficient α = 0.008 is used during dynamic simulations. The experimentally observed SW spectra (FFT of the time-resolved Kerr rotation data) match qualitatively with the simulated SW spectra. But due to some limitations in the micromagnetic simulations, we observe a slight quantitative disagreement between the experimental and simulated spectra [36]. As the triangular antidots have rounded corners, and hence suffer from small asymmetry in their shapes, the simulations have been performed by introducing the actual edge roughness of the triangular antidots. However, the precise edge roughness and deformation could not be reproduced by the finite difference method based micromagnetic simulations used here. The simulations have also been performed on similar antidot arrays after applying a two-dimensional periodic boundary condition (2D-PBC). The simulations with and without application of 2D-PBC show almost identical results. The simulation results with the introduction 2D-PBC are given in Supporting Information File 1.

**Figure 3:**
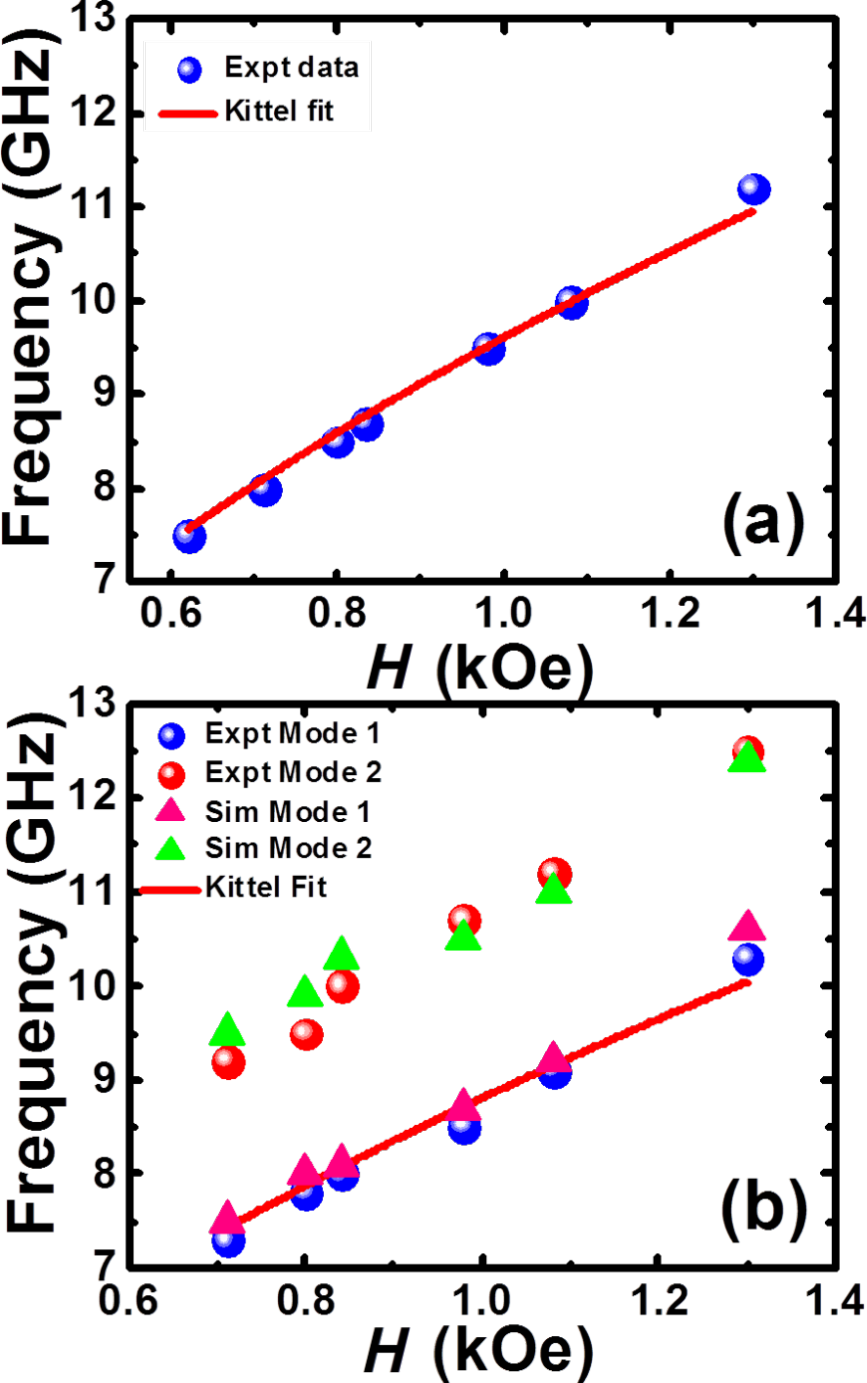
Bias magnetic field dependence of precessional frequencies of different SW modes for (a) Py blanket film and (b) antidot lattice S2 with lattice constant 700 nm.

Figure 3a shows the bias magnetic field dependence of the SW frequencies fitted with the Kittel formula for a 20 nm-thick Py blanket film. Figure 3b shows the same extracted from the experimental and simulated FFT spectra for S2. The experimental data points corresponding to mode 1 are well fitted with the Kittel formula. However, the higher frequency mode (mode 2) does not follow the same formula. The *M*_s_ value obtained from the Kittel fit of mode 1 is 712 emu cm^−3^, while the other magnetic parameters remain same as the Py blanket film. The difference between the simulated and experimental SW mode frequencies may arise due to random demagnetized regions at the edges and rounded corners of the triangular antidots, which is hard to precisely incorporate in the finite difference method based micromagnetic simulations such as OOMMF as used here. Further, we could not fit any of the experimental modes of S1 with the Kittel formula.

### Micromagnetic analysis of the collective magnetization dynamics in the arrays

**Figure 4:**
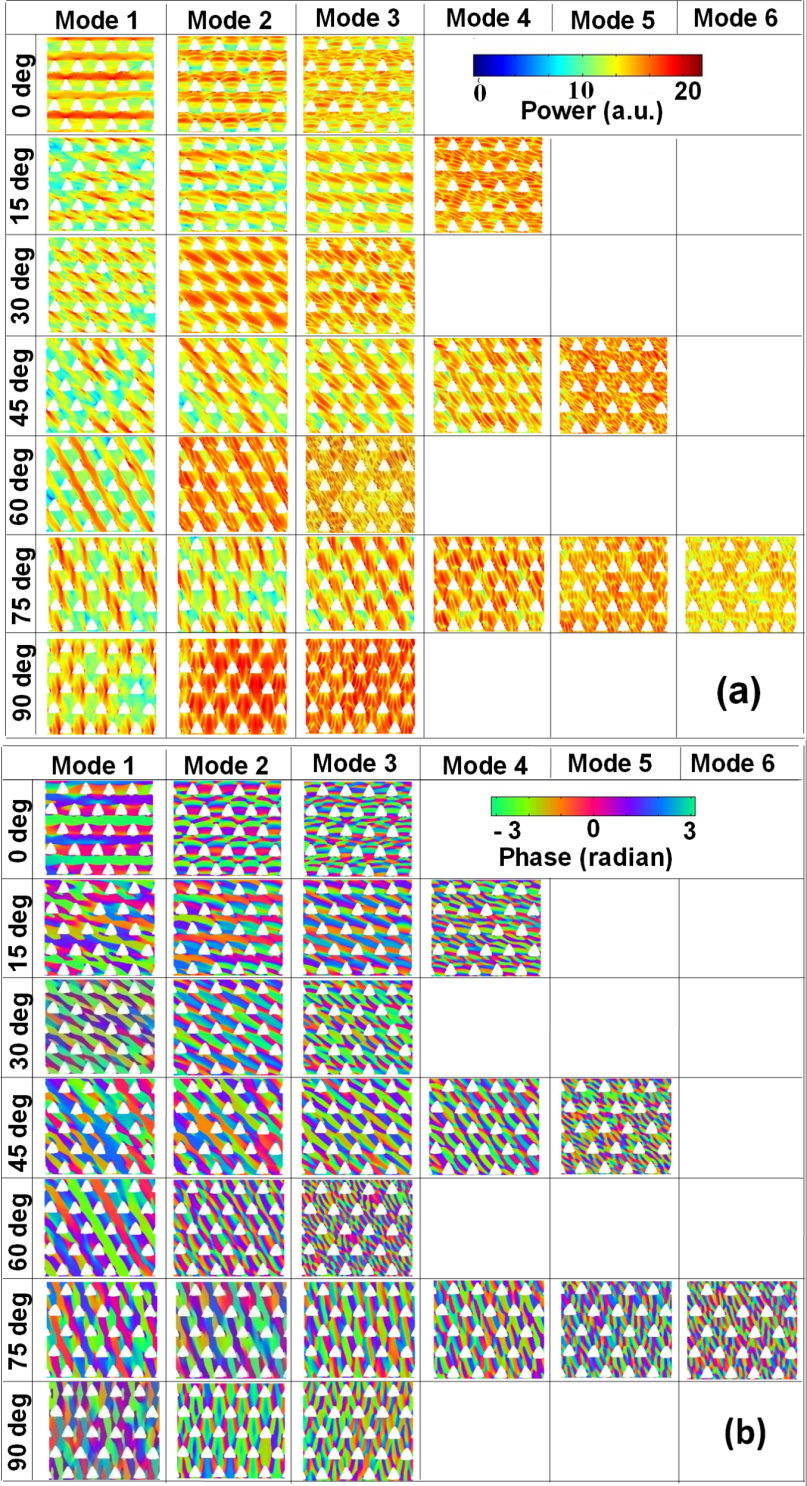
Spin-wave mode profile. (a) Power and (b) phase of S1 for different orientations of the in-plane bias magnetic field. The colour maps used for the mode profiles are shown inside the figure.

We have further simulated the spatial profiles of the resonant modes using a home-built code [40] and the simulated power and phase maps for S1 are shown in Figure 4a and Figure 4b, respectively. For φ = 0°, mode 1 has an extended character through the horizontal channels between the neighbouring antidot rows in the Damon-Eshbach (DE) geometry (i.e., extended in a direction orthogonal to the applied bias magnetic field). On the contrary, mode 2 is a localized mode where the highest spin-precession amplitude is localized in the same horizontal channels with quantization number *n* = 3. Mode 3 is again a quantized mode with higher quantization number (*n* = 5). When φ is rotated to 15°, mode 1 is an edge mode (EM) of the array and the highest spin-precession amplitude associated with this mode is found at the top vertex of each triangular hole. Interestingly, in the simulated profile, mode 2 for φ = 0° is split into two for φ = 15° (modes 2 and 3). These two modes are localized modes between diagonally situated next nearest neighbours but standing waves do not form exactly between the diagonally situated next nearest neighbours and become asymmetric due to asymmetry in the internal field profile. Mode 4 is the quantized mode with quantization number *n* = 5. When φ is further rotated to 30°, mode 1 is again an EM of the lattice with the highest spin-precession amplitude mainly concentrated due to the demagnetizing regions at the left-most vertex of each triangular antidot. Mode 2 is localized mode and as opposed to φ = 15°, here the standing wave is symmetric and forms exactly between the diagonally situated next nearest neighbours. Again, mode 3 is the quantized mode with *n* = 3. For φ = 45°, the lowest frequency mode is split into two modes (modes 1 and 2). For these two modes, overlap between localized modes generates a pseudo-extended mode through the channel marked by the dotted line shown in Figure 1a (diagonally extended channel). The next mode is again split into mode 3 and mode 4 and these two are localized modes along the same channel. Here, the highest frequency mode 5 is quantized with quantization number *n* = 5. Again, for φ = 60°, the spatial profiles of the SW spectra qualitatively match with that of φ = 0°. Here, mode 1 is a fully extended mode similar to that for φ = 0°, but the channel of propagation is different and it flows through the diagonally extended channel. Mode 2 is localized in the same channel with *n* = 3, and mode 3 is a quantized mode with higher quantization number (*n* = 7). Again, at φ = 75°, each mode is split into two. For the two lowest frequency modes (mode 1 and 2), the highest spin-precession amplitude is concentrated at the left-most vertex of the triangular antidots. These two are localized modes and seem to be running parallel through the diagonally extended channel of the array. The next higher frequency modes are quantized modes with quantization numbers increasing from mode 3 to mode 6. For φ = 90°, due to the unavailability of continuous channels along the vertical direction, mode 1 is again an EM of the lattice with the highest spin-precession amplitude mainly concentrated due to the demagnetizing regions at the left-most vertex of each triangular antidots. Mode 2 is a localized mode as compared to φ = 30° and here the standing wave is symmetric and forms exactly between the vertically situated next nearest neighbours. Mode 3 is again a quantized mode with higher quantization number (*n* = 5).

**Figure 5:**
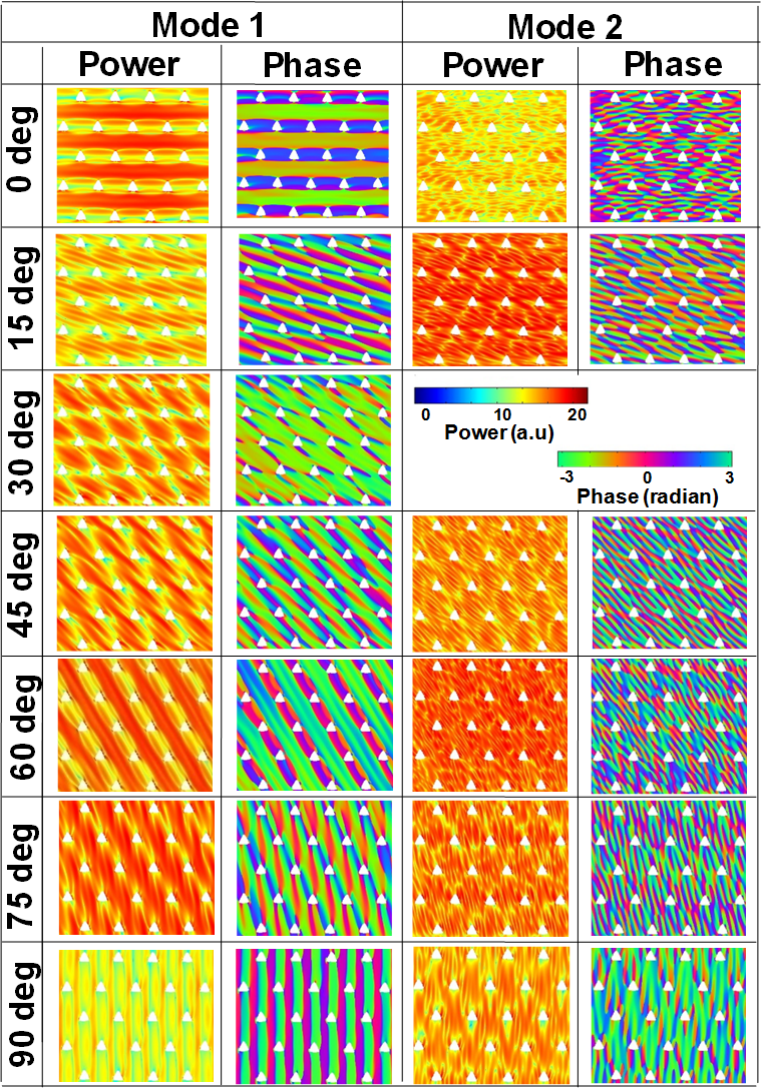
Spin-wave mode profile (power and phase) of S2 for different orientations of the in-plane bias field. The colour maps used for the mode profiles are shown inside the figure.

In S2, we observed a remarkable variation in the SW mode profiles as shown in Figure 5. In addition to horizontally and diagonally extended continuous channels, we also observed continuous channels in the vertical direction (which was unavailable in S1) and fully extended modes in DE geometry are obtained through the horizontal, diagonal and vertical channels for φ = 0°, 60° and 90°, respectively. For the other angles, the lower frequency extended, pseudo-extended or EMs (present in S1) are not present here and we observed localized and/or quantized modes.

### Magnetostatic field distribution of the arrays

**Figure 6:**
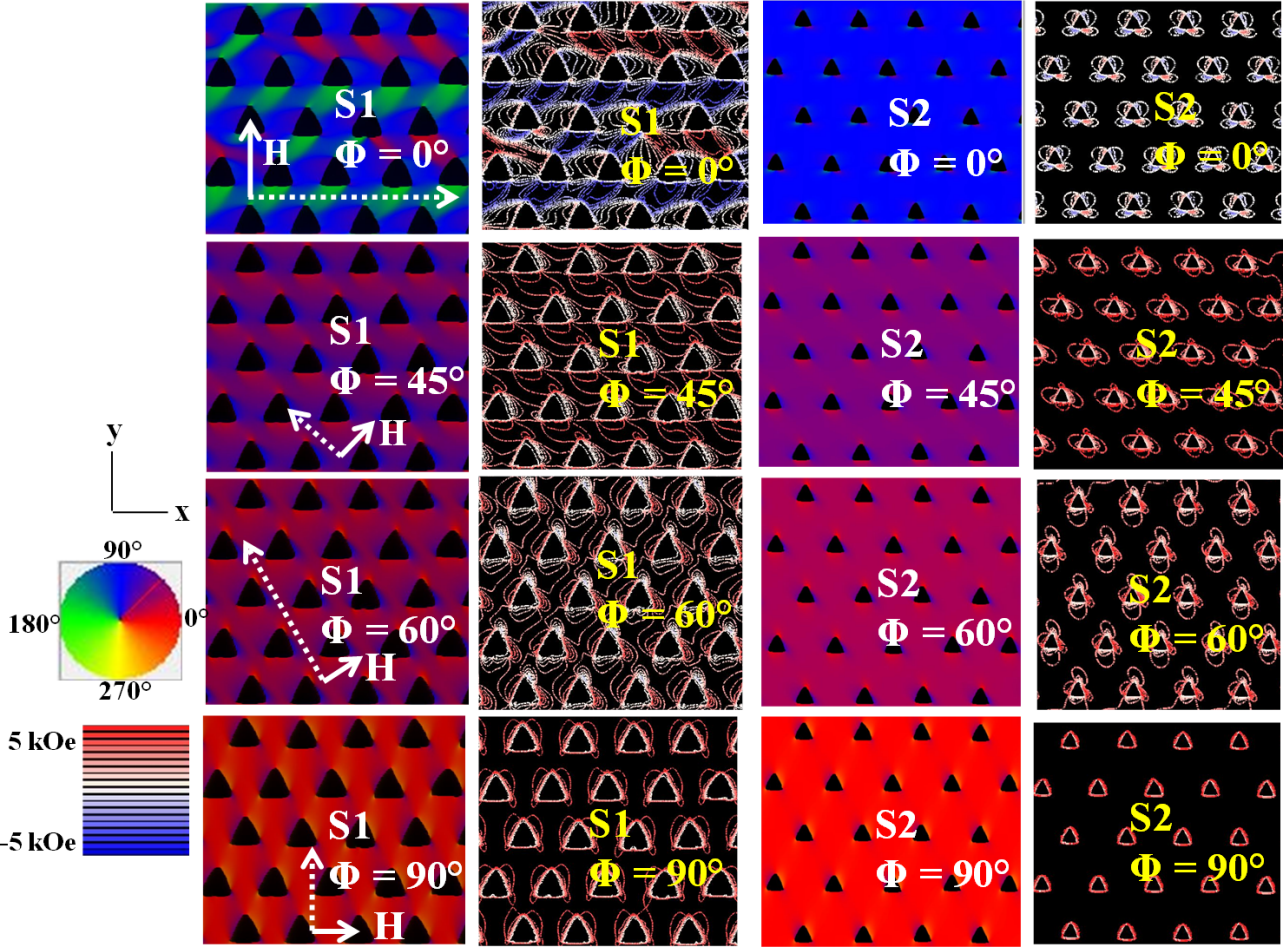
Magnetization distribution and the contour maps of simulated magnetostatic field distribution of the two arrays for specific orientations of the in-plane bias magnetic field obtained by micromagnetic simulations. The solid arrow lines indicate the direction of applied bias magnetic field and the dotted arrow lines (perpendicular to the direction of applied bias magnetic field) indicate the direction through which spin waves are supposed to propagate. Colour maps for both magnetization distribution and contour plot are shown in the left side of the figure.

We have further simulated the magnetostatic field distribution of the antidot arrays by using the LLG micromagnetic simulator [41]. Figure 6 shows the magnetization maps (domain plot) and the contour plots of simulated magnetostatic field distributions for the two arrays, at some specific orientations of the in-plane bias field.

This reveals the demagnetized regions and internal-field distribution around the antidots for different values of φ. For different φ-values, the surface charges at the boundaries between the antidots and magnetic layer lead to the formation of different types of domains through the demagnetization field. Due to the triangular shape of the antidots, the demagnetized regions, as well as magnetostatic field distributions around the arrays, are not symmetrical. As φ is varied, the domain structure and the internal field lines change considerably. This leads to the variation in the SW mode structures as well as the mode frequencies.

For S1 at φ = 0°, domains with magnetization reaching ±45° with respect to the y-axis (direction of applied bias magnetic field) are located within the regions between the base and the top apex of the vertically nearest neighbour triangular antidots. But due to the triangular shape of the holes and the small lattice constant, all the ±45° domains are not symmetrically situated. The domains with magnetization along the applied field are located within the central area of the unit cell. From the contour plot it is evident that the density of the internal field lines in the region between the base and top apex of vertically situated triangular holes (i.e., along the horizontal channel) is large. The hexagonal geometry gives the extended nature of the SW modes through the horizontal channel shown in Figure 1. For S1 at φ = 60°, the density of the internal field lines deceases along the horizontal channel but increases along the diagonal channel as small domains with magnetization directed nearly along y-axis are located at the left and right apex and domains with magnetization directed nearly along x-axis are located at the top-apex of each triangle. But along the diagonally extended channel, the magnetization points along the direction of applied field and SW shows an extended nature through this channel. Again, for S1 at φ = 45°, the density of the internal field lines is less compared to φ = 0° and 60°, along the horizontal and diagonal channels, respectively. Domains with magnetization directed nearly along the *x*-direction are located along the horizontal channel (between the base and top-apex of vertically situated nearest neighbours) and domains with magnetization directed nearly along the *y*-direction are located between horizontally situated nearest neighbours. But in this orientation of the bias field, the extended nature of SW is suppressed due to the absence of a channel along which only one type of domain could be observed. For S1 at φ = 90°, the density of the internal field lines reduces significantly and the demagnetizing regions become asymmetric around the triangular holes. In most of the regions, the magnetization points along the direction of the applied field. Only very small domains with magnetization pointing nearly along the *y*-axis are located at the corners of the triangular holes. But in this orientation, the extended nature of the SWs is not observed due to the hexagonal geometry of the lattice with a small lattice constant.

The domain structure changes when we consider the array S2 with a larger lattice constant. For φ = 0°, the asymmetric nature of the domain structure found in S1 is not observed in S2, all the domains almost coalesce, and very small ±45° domains are located only at the corners of the triangular holes for S2. In most of the regions, the magnetization points along the direction of the applied magnetic field and as the horizontal channels consist of only one type of domain, the power of the extended mode through this horizontal channel is considerably higher than that for S1. Similarly, in other orientations also, the domains coalesce more and only one type of domain is observed in most of the regions except for the triangular corners. Hence, in case of S2, we do not observe EMs as obtained in S1 and for all other orientations of the bias manetic field we observe either quantized or extended modes with comparatively higher power than S1.

## Conclusion

In conclusion we have investigated the effects of the orientation of the bias-magnetic field and lattice constant on the ultrafast magnetization dynamics and magnetostatic field distribution in a periodic array of triangular nanoholes forming a hexagonal antidot lattice in a thin Py film by using time-resolved Kerr microscopy. The experimental results reveal that the magnetization dynamics can be effectively tuned by the systematic variation of the orientation of the in-plane bias-magnetic field and lattice constant. Micromagnetic simulations successfully reproduced the experimental results and a fully extended SW mode is found to transform to quantized ones and vice versa simply by changing the in-plane orientation of the bias field. For the antidot lattice S1 (lattice constant 400 nm), the channels for SW propagation are found to be opened at φ = 0° and 60°. For φ = 45°, we observe a pseudo-extended nature of SW modes along the diagonally extended channel, whereas for the other angles, due to unavailability of continuous propagation channels, the powers of SWs are found to be concentrated at specific edges of the triangular holes. Interestingly, for S2 (lattice constant 700 nm), due to the increased inter-antidot separation, an additional SW propagation channel at φ = 90° gets opened. For other angles, the low-power edge modes are not present here due to the increased lattice constant, and for those angles, we mainly observe quantized and/or localized modes. The observed variation in the collective magnetization dynamics with the orientation of the in-plane bias field is attributed to the variation of the internal field distribution between the triangular-shaped antidots. The observed tunability of the magnetization dynamics and SW spectra with the variation in the orientation of the in-plane bias field and lattice constant is anticipated to be important for nanoscale magnonic crystal based technology.

## Experimental

### Fabrication

Two-dimensional arrays of Py antidots with triangular holes arranged in a hexagonal lattice have been fabricated by a combination of electron-beam lithography, electron-beam evaporation and ion milling. The 20 nm-thick Py film was deposited on a commercially available self-oxidized Si(100) substrate and a 60-nm-thick protective layer of Al_2_O_3_ was deposited on top of the Py film in an ultrahigh vacuum chamber at a base pressure of 2 × 10^−8^ Torr. The Al_2_O_3_ capping layer was deposited on the Py film to protect the samples from external contamination of the environment, degradation with time, and also from direct irradiation of laser light. A PMMA/MMA bilayer resist was used for electron-beam lithography to prepare the resist pattern on the Py thin film followed by argon ion milling at a base pressure of 1 × 10^−4^ Torr with a beam current of 60 mA for 6 min for etching out the Py film from everywhere except the unexposed resist pattern to create the triangular antidots.

### Measurement

A custom-built all-optical time-resolved magneto-optical Kerr effect (TRMOKE) microscope based on a two-colour collinear optical pump–probe geometry has been employed to measure the ultrafast magnetization dynamics of the antidot lattices. In this technique, the second harmonic (λ = 400 nm, fluence = 20 mJ/cm^2^, pulse width ≈100 fs, spot size = 1 μm) of the fundamental laser beam is generated by a second harmonic generator (SHG) from a mode-locked Ti:sapphire laser (Tsunami, Spectra Physics) to pump or excite the dynamics. The fundamental beam (λ = 800 nm, fluence = 5 mJ/cm^2^, pulse width ≈80 fs, spot size = 800 nm) placed at the centre of the pump beam is used to probe the dynamics of the sample by measuring the time-varying polar Kerr rotation from the sample. The magneto-optical Kerr rotation is measured by an optical bridge detector as a function of the time delay between the pump and probe beams. The pump and probe beams are spatially overlapped and focused together on the antidot lattice in a collinear fashion by using a single microscope objective (N.A. = 0.65). The sample is scanned by an *x*–*y*–*z* piezoelectric scanning stage, which gives high stability to the sample in the presence of feedback loops. The pump beam was chopped at 2 kHz frequency, and the phase-sensitive detection of the Kerr rotation and reflectivity were performed using lock-in amplifiers and an optical bridge detector at room temperature. A variable magnetic field is applied at a small angle (10°) to the sample plane and its in-plane component is defined as the bias magnetic field *H*. In the experiment, we effectively vary the azimuthal angle (φ) of *H* between 0° and 90° at intervals of 15° for the hexagonal antidot lattice by rotating the samples using a high-precision rotary stage while keeping the microscope objective and *H* constant. The pump and the probe beams are made to incident on the same region of the array for each value of φ.

## Supporting Information

File 1 Micromagnetic simulations of the antidot arrays by applying 2D-PBC.
