# Supplementary material for: Field-controlled ultrafast magnetization dynamics in two-dimensional nanoscale ferromagnetic antidot arrays
Source: Beilstein J Nanotechnol. 2018 Apr 9;9:1123–34. doi: 10.3762/bjnano.9.104 (PMC5905244; doi:10.3762/bjnano.9.104)
Supplement: File 1 — Micromagnetic simulations of the antidot arrays by applying 2D-PBC. [file Beilstein_J_Nanotechnol-09-1123-s001.pdf]

Supporting Information

for

Field-controlled ultrafast magnetization dynamics in two-dimensional nanoscale ferromagnetic antidot arrays

Anulekha De[1], Sucheta Mondal[1], Sourav Sahoo[1], Saswati Barman[2], Yoshichika Otani[3,4], Rajib Kumar Mitra[5] and Anjan Barman[1,*]

Address: [1]Department of Condensed Matter Physics and Material Sciences, S. N. Bose National Centre for Basic Sciences, Block JD, Sector III, Salt Lake, Kolkata 700 106, India, [2]Institute of Engineering and Management, Sector V, Salt Lake, Kolkata 700 091, India, [3]Institute for Solid State Physics, University of Tokyo, 5-1-5 Kashiwanoha, Kashiwa, Chiba 277-8581, Japan, [4]RIKEN-CEMS, 2-1 Hirosawa, Wako, Saitama 351-0198, Japan and [5]Department of Chemical Biological and Macro-molecular Sciences, S. N. Bose National Centre for Basic Sciences, Block JD, Sector III, Salt Lake, Kolkata 700 106, India

Email: Anjan Barman[*] - abarman@bose.res.in

[*] Corresponding author

Micromagnetic simulations of the antidot arrays by applying 2D-PBC

The simulation results with application of 2D-PBC show almost similar results as compared to those without the application of 2D-PBC. The simulations with 2D-PBC have been performed on the arrays with the introduction of actual edge deformation to the triangular antidots. The FFT power spectra of the antidot array S1, for φ = 0°, 45° and 60° are shown in Figure S1 and the corresponding power and phase profiles are shown in Figure S2a and Figure S2b, respectively. As compared to the spectra without the application of 2D-PBC, in this case also the spectra for φ = 0° and 60° are qualitatively similar in nature. In both cases we observe three modes and the number of modes increases to five for φ = 45°. However, the mode frequencies are slightly different as compared to the spectra without the application of 2D-PBC. For φ = 0°, mode 1 at 8.2 GHz has an extended character through the horizontal channels between the neighbouring antidot rows in the Damon-Eshbach (DE) geometry (i.e. extended in a direction orthogonal to the applied bias field). On the contrary, mode 2 at 10.2 GHz is a localized mode where highest spin-precession amplitude localized in the same horizontal channels with quantization number n = 3. Mode 3 at 11.5 GHz is again a quantized mode with higher quantization number (n = 5). Again, for φ = 60°, the spatial profiles of the spin waves qualitatively match with that of φ = 0°. Here, mode 1 at 7.5 GHz is a fully extended mode similar to that for φ = 0°, but the channel of propagation is different and it flows through the diagonally extended channel. Mode 2 at 10.2 GHz is localized in the same channel with n = 3, and mode 3 at 13.8 GHz is quantized mode with higher quantization number (n = 7). For φ = 45°, the lowest frequency mode is split into two modes (mode 1 at 8.1 GHz and mode 2 at 8.7 GHz). For these two modes, overlap between localized modes generates a pseudo-extended mode through the diagonally extended channel. The next mode is again split into modes 3 and 4 (at 9.8 GHz and 10.5 GHz, respectively)

and these two are localized modes (n = 3 for mode 3 and n = 5 for mode 5) along the same channel. Here, the highest frequency mode 5 at 12.2 GHz has a quantized nature with quantization number n = 7.

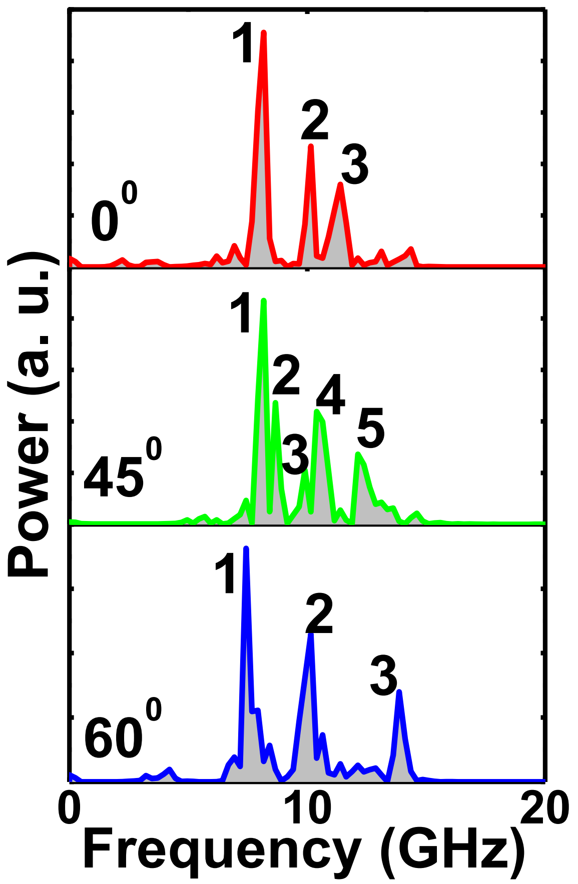

Figure S1: FFT power spectra of the simulated time-domain magnetization of the antidot array S1 after the application of 2D-PBC for bias field applied along φ = 0°, 45° and 60°.

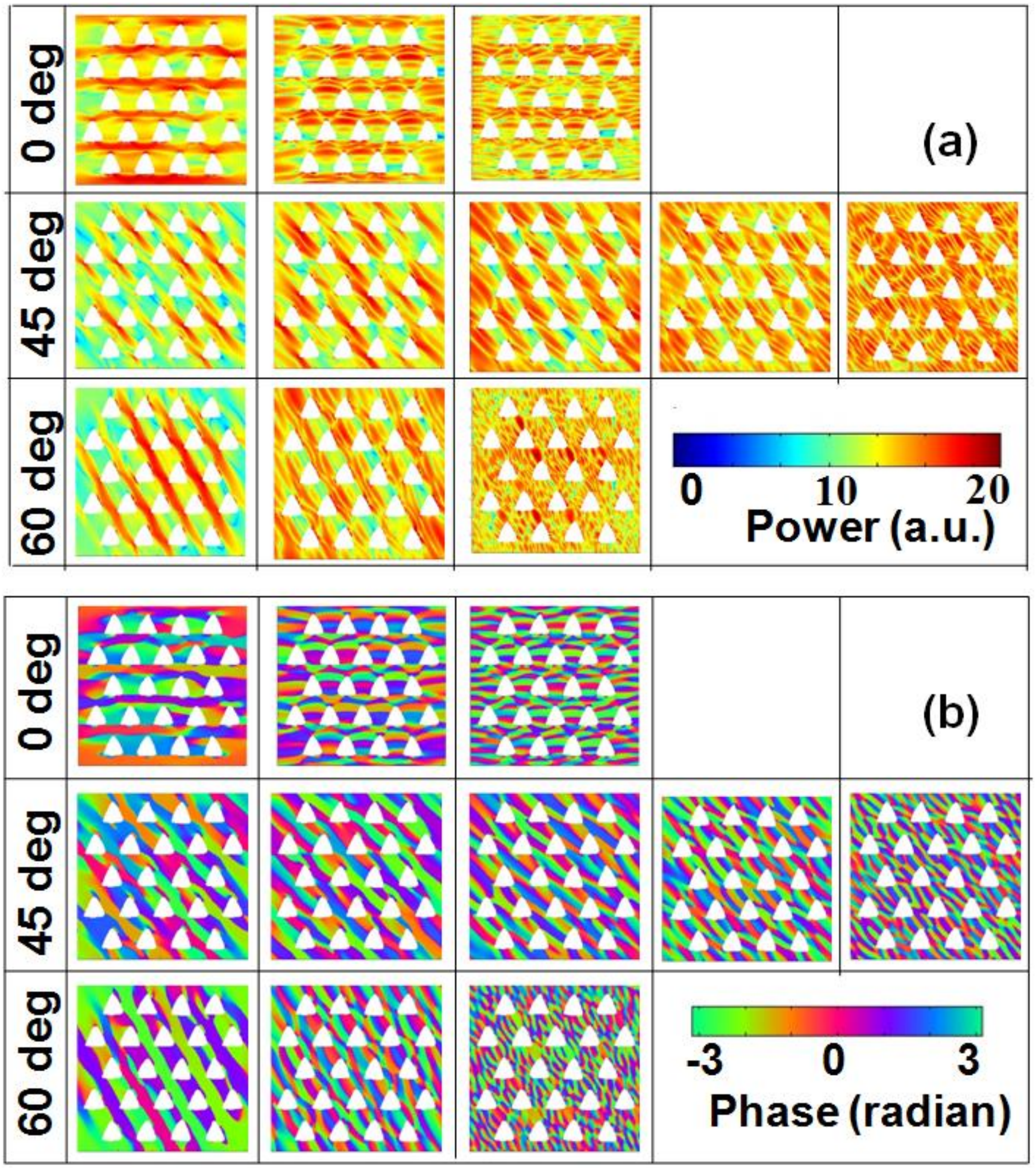

Figure S2: Spin-wave mode profiles: (a) power and (b) phase of S1 after the application of 2D-PBC for bias field applied along φ = 0°, 45° and 60°. The colour maps used for the mode profiles are shown inside the respective figures.
